# Oligomerization of Mutant p53 R273H is not Required for Gain-of-Function Chromatin Associated Activities

**DOI:** 10.3389/fcell.2021.772315

**Published:** 2021-11-22

**Authors:** George K. Annor, Nour Elshabassy, Devon Lundine, Don-Gerard Conde, Gu Xiao, Viola Ellison, Jill Bargonetti

**Affiliations:** ^1^ The Department of Biological Sciences Hunter College, Belfer Research Building, City University of New York, New York, NY, United States; ^2^ The Graduate Center Biology and Biochemistry Programs of City University of New York, New York, NY, United States; ^3^ Department of Cell and Developmental Biology, Weill Cornell Medical College, New York City, NY, United States

**Keywords:** mutant p53, oligomerization domain, gain-of-function, chromatin, replication-associated

## Abstract

The *TP53* gene is often mutated in cancer, with missense mutations found in the central DNA binding domain, and less often in the C-terminal oligomerization domain (OD). These types of mutations are found in patients with the rare inherited cancer predisposition disorder called Li-Fraumeni syndrome. We previously found that mutant p53 (mtp53) R273H associates with replicating DNA and promotes the chromatin association of replication-associated proteins mini-chromosome maintenance 2 (MCM2), and poly ADP-ribose polymerase 1(PARP1). Herein, we created dual mutants in order to test if the oligomerization state of mtp53 R273H played a role in chromatin binding oncogenic gain-of-function (GOF) activities. We used site-directed mutagenesis to introduce point mutations in the OD in wild-type p53 (wtp53), and mtp53 R273H expressing plasmids. The glutaraldehyde crosslinking assay revealed that both wtp53 and mtp53 R273H formed predominantly tetramers, while the single OD mutant A347D, and the dual mtp53 R273H-A347D, formed predominantly dimers. The R337C, L344P, mtp53 R273H-R337C, and mtp53 R273H-L344P proteins formed predominantly monomers. Wtp53 was able to activate the cyclin-dependent kinase gene *p21/waf* and the p53 feedback regulator *MDM2*. As expected, the transactivation activity was lost for all the single mutants, as well as the mtp53 R273H-dual mutants. Importantly, mtp53 R273H and the dual oligomerization mutants, R273H-A347D, R273H-R337C, and R273H-L344P were able to interact with chromatin. Additionally, the dual oligomerization mutants, R273H-A347D, R273H-R337C, and R273H-L344P, maintained strong interactions with MCM2 and PARP1. Our findings suggest that while mtp53 R273H can form tetramers, tetramer formation is not required for the GOF associated chromatin interactions.

## Introduction

Cancers often have genetic mutations in the *TP53* gene that can be both inherited and spontaneous (Levine, 2021). These mutations often disrupt the sequence-specific DNA binding activity of wild-type p53 (wtp53) and can also be found, albeit less frequently, in the C-terminal oligomerization domain (OD) (Levine, 2021). A subset of *TP53* mutations transform the gene into an oncogene, producing a class of mutant p53 (mtp53) proteins known to have gain-of-function (GOF) properties that help in tumor promotion (Weisz et al., 2007; Sullivan et al., 2018). The GOF mtp53 proteins possess biochemical properties distinct from wtp53, including longer half-lives, transcriptional activation of non-canonical p53 target genes, and exhibit stable complex formation with both canonical and non-canonical protein interaction partners (Sabapathy, 2015; Sabapathy and Lane, 2018). Amino acids in the central region of wild-type p53 form direct DNA contacts and conformationally coordinate the protein for stable sequence-specific interactions at p53 response elements; the hotspot cancer amino acid mutations in Arg 248 (R248) and Arg 273 (R273) correspond to amino acid residues that make direct contact with the DNA backbone (Cho et al., 1994). The fact that p53 functions as a tetramer was discovered in part by glutaraldehyde cross-linking experiments that examined the entire protein (Friedman et al., 1993). Crystal structure studies exclusively with the OD further clarified how critical p53 amino acids affect tetramer formation (Friedman et al., 1993; Jeffrey et al., 1995).

The OD of p53 is composed of amino acid residues stretching from position 323-355 that assist in sequence specific DNA binding and transcriptional activation (Waterman et al., 1995). The OD of p53 is comprised of a ß-strand (Glu326-Arg333), a tight turn (Gly334), and an α-helix (Arg335-Gly355) (Jeffrey et al., 1995; Kamada et al., 2016). Two monomeric p53s form a dimer when the ß-strands of their ODs interact in an anti-parallel manner. This anti-parallel ß-strand-interaction allows each dimer to have its α-helix projecting outward, allowing it to form a dimer-of-dimers with other α-helices to form a four-helix tetramer (Johnson et al., 1995). The four-helix tetramer configuration positions the DNA-binding domain close to the p53 response element for easy interaction and is required for transactivation of target genes (Weisz et al., 2007; Goh et al., 2011).

Tetramerization of wtp53 is important for tumor-suppressor activities that lead to the activation of apoptosis (Fischer et al., 2016). Compromising the ability of wtp53 to form a tetramer downregulates the expression of p53 target genes (Kawaguchi et al., 2005). In Li-Fraumeni syndrome (a condition where patients present with germline p53 mutations and have a predisposition to early-life cancer development), point mutations within the OD (R337C, A347D, or L344P) destabilize tetramer formation and decrease the ability of p53 to bind DNA, as well as activate transcription of *p21*, *Bax,* and *PUMA* (Jeffrey et al., 1995; Davison et al., 1998; Lomax et al., 1998). Interestingly, in cancers the p53 associated mutations have never been reported to occur simultaneously in the OD and in the DNA binding domain. This suggests that the OD and DNA binding domain mutations make independent contributions to p53 transactivation activity. Therefore there is no selection for dual mutants in cancers. Simply inhibiting the transcription factor function of p53, in one way or the other, would be enough to promote tumorigenesis. However, p53 is known to have functions that are separable from its transcription factor activity. For example, p53 participates in the regulation of DNA replication (Bargonetti and Prives, 2019). It may be some of these functions that are co-opted by different GOF mtp53 proteins. Recently, a C-terminal frame shift p53 mutant has been shown to gain some new functions (Tong et al., 2021). We found that mtp53 R273H associates with replicating chromatin, but it was not clear if the oligomerization of mtp53 played a role in chromatin-association (Xiao et al., 2020). With this in mind, we decided to create dual mutants (even though these do not exist in cancers). This was done in order to investigate whether the oligomerization state of mtp53 R273H influenced the DNA binding of mtp53 R273H, and the GOF associated replication activities.

GOF mtp53 R273H interacts with replicating DNA, and the replication associated proteins poly ADP-ribose polymerase 1 (PARP1), and the DNA helicase mini-chromosome maintenance complex (MCM2-7) in a mtp53-PARP-MCM axis (Polotskaia et al., 2015; Qiu et al., 2017; Bargonetti and Prives, 2019; Xiao et al., 2020). The mtp53-PARP-MCM axis on chromatin suggests a role for GOF mtp53 in DNA repair and/or replication mechanisms. Exogenous expression of GOF mtp53 (R175H or R273H) in human cells correlates with the increased transcription of DNA replicating factor *CDC7* (which increases DNA replication origin firing) (Datta et al., 2017). The transcriptional activation of previously silent genes in the presence of mtp53 in cancer cells has recently been associated with p53 mutations driving aneuploidy, rather than as a direct transcriptional response (Redman-Rivera et al., 2021).

Li-Fraumeni syndrome (LFS) predisposes patients to early onset of different types of cancer (Srivastava et al., 1990; Malkin, 1993; Malkin, 2011). In a cohort in Brazil, it was reported that the R337C mutation in the OD of p53 predisposes LFS patients to adenocarcinoma (Fischer et al., 2018). While it has already been reported that mutations in the OD region compromise wtp53 transcriptional activity, we wanted to determine if LFS associated OD mutations changed mtp53 R273H chromatin-associated activities. We used site-directed mutagenesis to introduce point mutations in plasmids expressing either wtp53 or mtp53 R273H to alter amino acids R337C, A347D, and L344P. We observed that exogenously expressed mtp53 R273H formed tetramers, and that dual mtp53 R273H-A347D formed predominantly dimers, while dual mtp53 R273H-R337C and R273H-L344P formed predominantly monomers. The destabilizing oligomerization mutations to create dual-mtp53 did not inhibit mtp53 R273H interactions with chromatin. Moreover, the interaction between mtp53 R273H and MCM2 or PARP1 were maintained in the dual-R273H oligomerization mutants. These findings suggest that oligomerization of GOF mtp53 R273H does not significantly influence GOF chromatin associated activities.

## Materials and Methods

### Materials

Solvents and chemicals including DMSO, glutaraldehyde, temozolomide, and talazoparib were obtained from Sigma-Aldrich (St. Louis, MO, United States). DO1 p53 (Cat# sc-126) monoclonal and PARP1 (Cat# sc-7150) rabbit polyclonal antibodies were purchased from Santa Cruz (United States). MCM2 (Cat# 12079s) mouse antibody was purchased from Cell Signaling Technology. An Eppendorf 5,415 refrigerated centrifuge was used for preparation of all extracts.

### Cell Culture and Drug Treatments

Human breast cancer cell line MDA-MB-468 was purchased from ATCC (www.atcc.org) and the HCT116 colon cancer cell line that is p53−/− was a gift from Bert Vogelstein and were made as described (Bunz et al., 1998; Bunz et al., 1999). Cell lines were regularly authenticated via short tandem repeat technology (Genetica DNA Laboratories). Cells were routinely checked for mycoplasma contamination by PCR assay (ATCC). Fresh cells were thawed when the passaging period was around 30. Cells were maintained at 5% CO_2_ in a 37°C humidified incubator. HCT116 p53−/− and MDA-MB-468 cells were cultured in McCoy’s 5A (Gibco) and DMEM media (Corning) respectively, with 50 U/ml penicillin, 50 μg/ml streptomycin (Mediatech), 5 ug/ml plasmocin (InvivoGen) and supplemented with 10% FBS (Gemini). PARP-dependent recruitment of proteins to chromatin was assayed by exposure of HCT116 cells to 1 mM Temozolomide (Sigma-Aldrich; 100 mM stock solution in DMSO) and 10 μM Talazoparib (Selleckchem; 20 mM stock solution in DMSO) combination treatment for 4 h at 37°C followed by chromatin isolation as described (Qiu et al., 2017).

### Site-Directed Mutagenesis, Clone Validation, and Transfection

To generate clones expressing p53 with the desired mutations in the oligomerization domain, we used the NEBasechanger (https://nebasechanger.neb.com) platform to design primers to introduce specific point mutations within the OD of pCMV-FLAG-wtp53 and pCMV-FLAG-p53R273H plasmids which express wtp53 or R273H mtp53 (Hamard et al., 2012). For R337C, the primer pair was F: TGG​GCG​TGA​GtG​CTT​CGA​GAT and R: CGG​ATC​TGA​AGG​GTG​AAA​TAT​TCT​C (annealing temperature 66°C). For A347D, the primer pair was F: CTG​AAT​GAG​GaC​TTG​GAA​CTC and R: CTC​TCG​GAA​CAT​CTC​GAA​G (annealing temperature 62°C). Lastly, for L344P the primer pair was F: TTC​CGA​GAG​CcG​AAT​GAG​GCC and R: CATCTCGAAGCGCTCACG (annealing temperature 65°C). A PCR reaction was set up with CMV-wtp53 or CMV-R273H plasmid template and the Q5 Hotstart high-fidelity 2X master mix (NEB) at the specific annealing temperature of each primer pair. After confirming PCR amplicon with agarose gel electrophoresis, a kinase-ligase-Dpn1 (NEB) reaction was done before transforming DH5α competent cells. DNA was isolated from cultured transformants (grown in LB+50 μg/ml Amp) using the Qiagen miniprep/midiprep kit, sequenced (Genewiz) using a p53 Exon 8 F primer 5′ ACA​GCA​CAT​GAC​GGA​GGT​TGT, and plasmids harboring mutations were confirmed using the Benchling™ platform. Sequences were compared to TP53 cDNA from the GRCh38 homo sapien reference genome using the benchling platform’s external databases. Plasmids with the desired OD mutations were transfected into HCT116 p53−/− cells using either the lipofectamine™ (Invitrogen) or the electroporation-based Neon Transfection System™ (ThermoFisher) as directed by the manufacturers. For the nucleofection, the transfection protocol as previously described for MDA-MB-468 cells was followed with little modification (Ellison et al., 2021). The conditions for introduction of plasmids into HCT116 p53−/− cells (7 μg/1,000,000 cells) using the Neon were pulsation 1x for 20 ms at 1530 V followed by culturing in McCoy’s 5A media +10% FBS without antibiotics.

### Glutaraldehyde Chemical Cross-Linking Assay

This assay was carried out as described previously for MDA-MB-468 cells and purified R273H mtp53 (Ellison et al., 2021; Xiao et al., 2021). Cells were washed with cold PBS, harvested by scraping in cold PBS, and centrifuging at 1,400 *g* (1,100 rpm) for 7 min. Harvested cells were lysed with phosphate lysis buffer (1x PBS, 10% glycerol, 10 mMEDTA, 0.5% NP-40, 0.1M KCl, 1 mM PMSF, 8.5 μg/ml aprotinin, 2 μg/ml leupeptin, and phosphatase inhibitor cocktail). A total of 50 μg of protein lysates were treated without, or with, glutaraldehyde at a final concentration of 0.005%. The lysates were then incubated at room temperature for 20 min on a shaker. The crosslinking was stopped by the addition of 1/6 of the volume with 6X protein sample buffer (6X SDS Laemmli sample buffer, 0.2 M DTT) and heated at 95°C for 10 min 25 μg was resolved on an 8% SDS-PAGE gel and Western blot analysis was performed with DO1 p53 monoclonal antibody.

### TaqMan Real-Time PCR

Total RNA was isolated from cell cultures as dictated by the experimental design using the Qiagen RNeasy™ kit as directed by the manufacturers (www.qiagen.com/HB-0435). The Applied Biosystems High-Capacity cDNA Reverse Transcription kit was used to generate cDNA from 5 μg of total RNA from each cell sample. The relative abundance of the *TP53, p21, MDM2, RRM2* and *CDC7* mRNA within each cell was measured by quantitative-PCR using the Applied Biosystems TaqMan real-time PCR with FAM dye-labeled probes and *GAPDH* as an endogenous control (Thermofisher Scientific *TP53* ID# 01034249_m1*, p21* cat# 4331182, *MDM2* cat# 4351372 *RRM2* cat# 01072069_g1, *CDC7* ID# 00177487_m1, and *GAPDH* ID# 02786624_g1) and the Applied Biosystems QuantStudio seven Flex instrument.

### Whole-Cell Lysis and Immunoblotting Assay

Cells were harvested by low speed centrifugation at 1,400 *g* (1,100 rpm) for 7 min at 4°C. Cells were washed three times with ice-cold PBS and resuspended in RIPA buffer (0.1% SDS, 1% IGEPAL NP-40, 0.5% Deoxycholate, 150 mM NaCl, 1 mM EDTA, 0.5 mM EGTA, 50 mM Tris-Cl pH 8.0, 1 mM PMSF, 8.5 μg/ml Aprotinin, 2 μg/ml Leupeptin and phosphatase inhibitor cocktail). The cell suspension was incubated on ice for 30 min to lyse the cells, with gentle vortexing every 5 min, after which, lysates were subject to sonication 3x for 30 s pulses/30 s rest on ice at 98% amplitude and then centrifuged at 15,700 *g* (13,200 rpm) for 30 min at 4°C. The protein concentrations of clarified cell extracts were determined using the Bradford assay (Bio-Rad), and 50 μg of extracts were analyzed for specific proteins by electro-transfer onto Polyvinylidene fluoride membrane (Amersham-GE Biosciences) following SDS-PAGE. The membrane was blocked with 5% non-fat milk (Bio-Rad) in either 1X PBS-0.1% Tween-20 or 1X TBS-0.1% Tween-20 followed by an overnight incubation with primary antibody at 4°C. The membrane was washed 3x with either 1X PBS-0.1% Tween-20 or 1X TBS-0.1% Tween-20 and incubated with Cy5-and Cy3-linked secondary antibodies (Amersham Biosciences) for 1h at room temperature. The signal was detected with the Typhoon FLA 7000 laser scanner (GE Healthcare). Primary antibodies used were; 1) anti-p53 DO1 p53 (Santa Cruz Cat# sc-126), 2) anti-PARP1 (Santa Cruz Cat# sc-7150), 3) anti-MCM2 1E7 (Cell Signalling Technology Cat# 12079s).

### Chromatin Fractionation Assay

Localization of mtp53 proteins to chromosomes was assessed using a version of the Chromatin Fractionation Assay as described (Ellison et al., 2021). Cells were harvested 24 h post-transfection as dictated by experimental conditions by scraping, pelleted, and washed with ice-cold PBS three times. The pellet was resuspended in Buffer A (10 mM HEPES, 10 mM KCl, 1.5 mM MgCl_2_, 300 mM Sucrose, 1 mM DTT, 10% Glycerol, 0.1 mM PMSF, 1 μg/ml Leupeptin, 1 μg/ml Pepstatin A, and 2 μg/ml Aprotinin) with 0.1% Triton X-100 using 3X the pellet volume. The resuspended pellet was centrifuged at 1,500 *g* (4,000 rpm) for 5 min after incubating on ice for 5 min. The resulting pellet containing nuclei was saved and the supernatant spun at 15,700 *g* (13,200 rpm) for 5 min; the supernatant from this centrifugation step was saved as S1. The nuclei from each sample were washed twice in Buffer A+ 0.15% Triton X-100 then lysed in Buffer B (3 mM EDTA, 0.2 mM EGTA, 1 mM DTT, 0.1 mM PMSF, 1 μg/ml Leupeptin, 1 μg/ml Pepstatin A, and 2 μg/ml Aprotinin) on ice for 30 min. The chromatin for each sample was separated from the nuclear lysate (S2) by centrifugation for 4 min at 1,500 *g* (4,000 rpm) at 4°C, washed with Buffer B, and then collected by centrifugation as described above. The chromatin pellet was resuspended in Buffer B and sonicated on ice to shear genomic DNA, the resulting solution was saved as the chromatin fraction. The protein concentrations were measured using the Bradford assay (Bio-Rad). ImageJ quantification is shown in Supplementary Figure S3A. The ImageJ software was used to determine the western blot band intensity with signals normalized to their respective Lamin control. The relative expression of p53 in the HCT116−/− transfected cells was determined by using the level of R273H protein in the no drug treatment as the reference sample. The relative expression of MCM2 and PARP1, was determine by using the protein levels in the empty vector transfection sample with no drug treatment as the reference samples.

### Detergent Solubility Assay

The method was derived from a DNA repair tight tethering detergent assay (Iwabuchi et al., 2003). Transfected HCT116 p53−/− cells were harvested and then stored at −80°C. For preparation of the soluble and insoluble fractions, the frozen cell pellets were lysed for 1 h at 4°C in TNE (pH 8.0) buffer (50 mM Tris, pH8.0,120 mM NaCl, 1 mM EDTA, 0.5% Nonidet P-40, 100 g/ml phenylmethylsulphonyl fluoride, 5 g/ml aprotinin, 5 g/ml Pepstatin, 2 g/ml leupeptin, 50 mM NaF, 2 mM Na₃VO₄·2H₂O (Sodium Orthovanadate)). Approximately 3x the cell pellet volume of TNE buffer was used for lysis. The lysate was divided into the soluble and insoluble fractions by centrifugation at 15,700 *g* (13,200 rpm) for 10 min at 4°C. The supernatant-soluble fraction was transferred to a new tube. The remaining pellet, or insoluble fraction was resuspended in PBS containing 2% SDS (∼5x insoluble cell pellet volume) and then sonicated 3 times for 30 s pulses/30 s rest on ice at 98% amplitude (QSonica, LLC Q700). The protein concentration for the soluble fraction was measured using the Bradford assay, while the protein concentration of the insoluble fraction was obtained by absorbance at 280 nm reading from Nanodrop Spectrophotometer. Samples were analyzed by western blotting as described above. ImageJ quantification is shown in Supplementary Figure S3B and determined as described above for chromatin fractionation samples.

### Immunofluorescent Assay Coupled Proximity Ligation Assay

The protocol for the proximity ligation assay using the Sigma Aldrich Duolink Kit™ (Cat # DUO92008) was as described previously (Xiao et al., 2020). Cells were seeded in a 12-well glass-bottomed plate at 1 × 10^5^ cells per well in complete McCoy 5A media supplemented with 10% FBS without antibiotics. After 24 h, 1.6 ug of plasmid DNA was added to each well via the lipofectamine transfection system as described by the manufacturer. After 24 h post-transfection, the media was removed, and the cells were washed with cold PBS 3 times. The cells were then fixed in 4% formaldehyde for 15 min and permeabilized in 0.5% Triton X-100 for 10 min at room temperature. The Duolink *In-situ* Red Kit (Cat # DUO92101) was used for the PLA. Blocking buffer was added to each well and incubated in a humidified chamber at 37°C for 30 min. After aspirating the blocking buffer, primary antibodies were added to each well and incubated in a humidified chamber overnight at room temperature. Wash buffer A (cat # DUO82049) was used to wash the cells 3 times for 5 min each. The PLA Plus/Minus secondary antibody probes were added to the wells and incubated in a humidified chamber for 60 min at 37°C. After that, the cells were washed 2 times with buffer A for 2 min each. The ligation step was performed for 30 min incubation at 37°C, washed 2 times with buffer A and the amplification was done for 100 min at 37°C. The wells were washed with buffer B for 10 min and incubated with a FITC-labelled secondary antibody to detect p53. After washing 3 times, mounting media containing DAPI was added to each well, rocked for 15 min, and images were taken using the Nikon A1 confocal microscope. Images obtained were processed with the Nikon NIS Element software, ImageJ and Cellprofiler. The foci/cell were plotted, and statistical analysis was performed in GraphPad Prism 9.

### Statistical Analysis

Statistical analyses were performed using GraphPad Prism 9. Results are expressed as mean +SEM. Statistical significance for hypothesis testing was performed by a one-way ANOVA with multiple comparison. The following format was used to assign significance based on *p*-value: **** represents a *p*-value ≤ 0.0001 and ns represent non-significant.

## Results

### p53 Tetramer Formation Is Destabilized by Introducing Mutations in the OD

**FIGURE 1:**
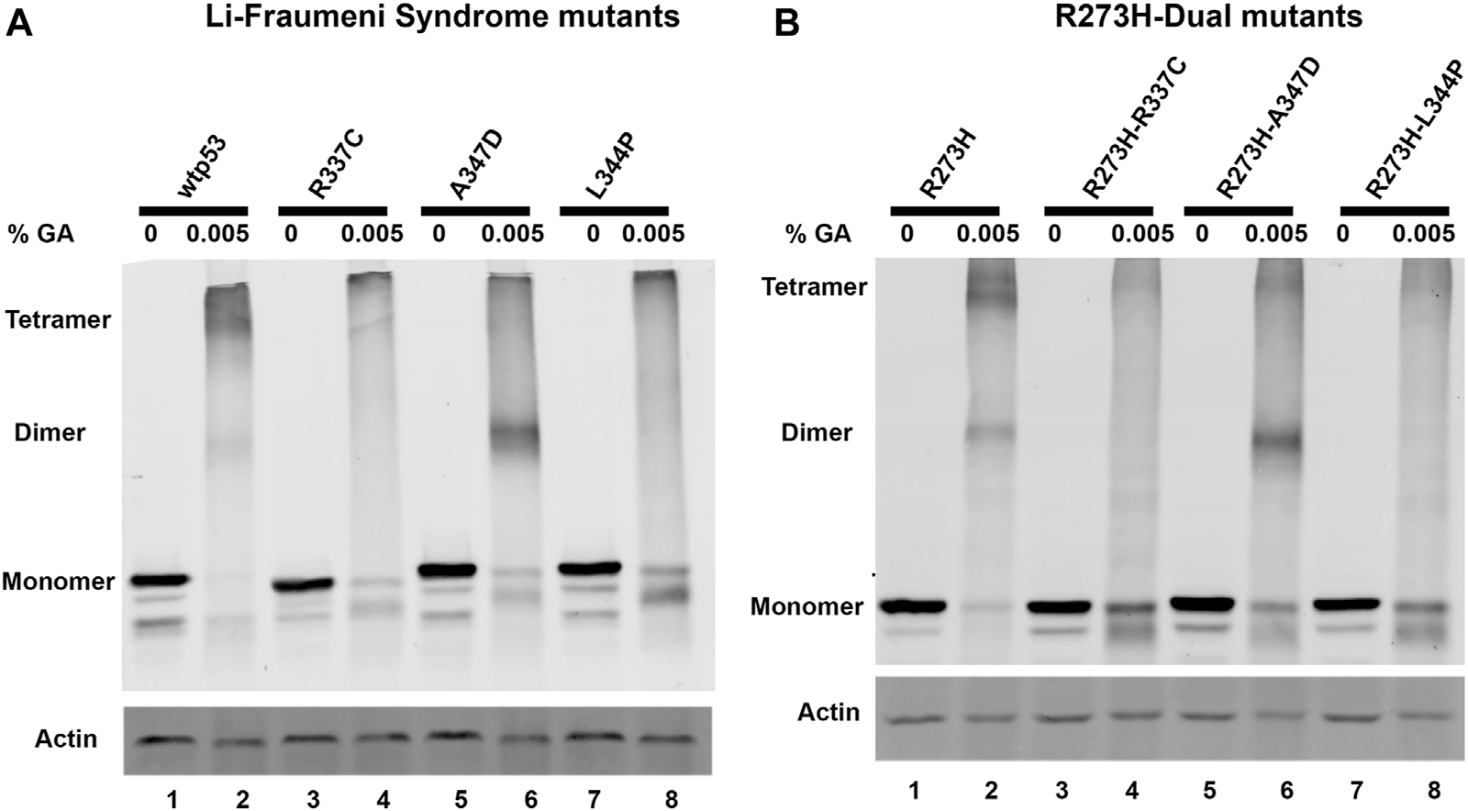
R273H-dual mtp53 mutants destabilize oligomerization of mtp53 R273H similar to Li-Fraumeni Syndrome single mutants. HCT116 p53−/− cells were transiently transfected with plasmids expressing either **(A)** wtp53, or single mutants R337C, A347D or L344P; or **(B)** mtp53 R273H, or dual mutants R273H-R337C, R273H-A347D, or R273H-L344P. After 24 h post transfection, cells were pelleted, and lysate prepared. 50 μg of the resulting cell lysates were treated with either 0% (lanes 1,3,5,7) or 0.005% (lanes 2,4,6,8) glutaraldehyde for 20 min at room temperature. Samples were run on an 8% SDS-PAGE and oligomerization determined by western blotting using anti-p53 DO1 antibody. Actin was used as a normalizer and showed very minor shift in mobility with 0.005% glutaraldehyde. Data presented was reproduced in three biological replicates.

We used site-directed mutagenesis to introduce oligomerization mutations to change p53 amino acid residues R337C, A347D, or L344P in plasmids for exogenous expression of either wtp53 or mtp53 R273H. When the Li-Fraumeni OD-specific associated point mutations were introduced individually, or as dual mutants within mtp53 R273H, they destabilized tetramer formation (Figures 1A,B). The exogenously expressed wtp53 and mtp53 R273H proteins predominantly formed dimers and tetramers when 0.005% glutaraldehyde was added (Figures 1A,B, lanes 1, 2), whereas both single A347D and dual R273H-A347D polypeptides shifted to form predominantly dimers (Figures 1A,B, lanes 5, 6). The Li-Fraumeni mutations R337C and L344P, as well as the dual mutants R273H-R337C and R273H-L344P, were unable to form dimers or tetramers (Figures 1A,B, lanes 3–8). We also confirmed that endogenous mtp53 in many different human breast cancer cell lines formed tetramers (Supplementary Figure S1). These data indicate that mtp53 can exist as tetramers in human cells, and that we successfully disrupted mtp53 R273H oligomerization in the R273H R337C, A347D, or L344P dual mutants.

### Transactivation of *p21* and *MDM2* Is Activated by wtp53, but Not mtp53 R273H, or OD Mutants

**FIGURE 2:**
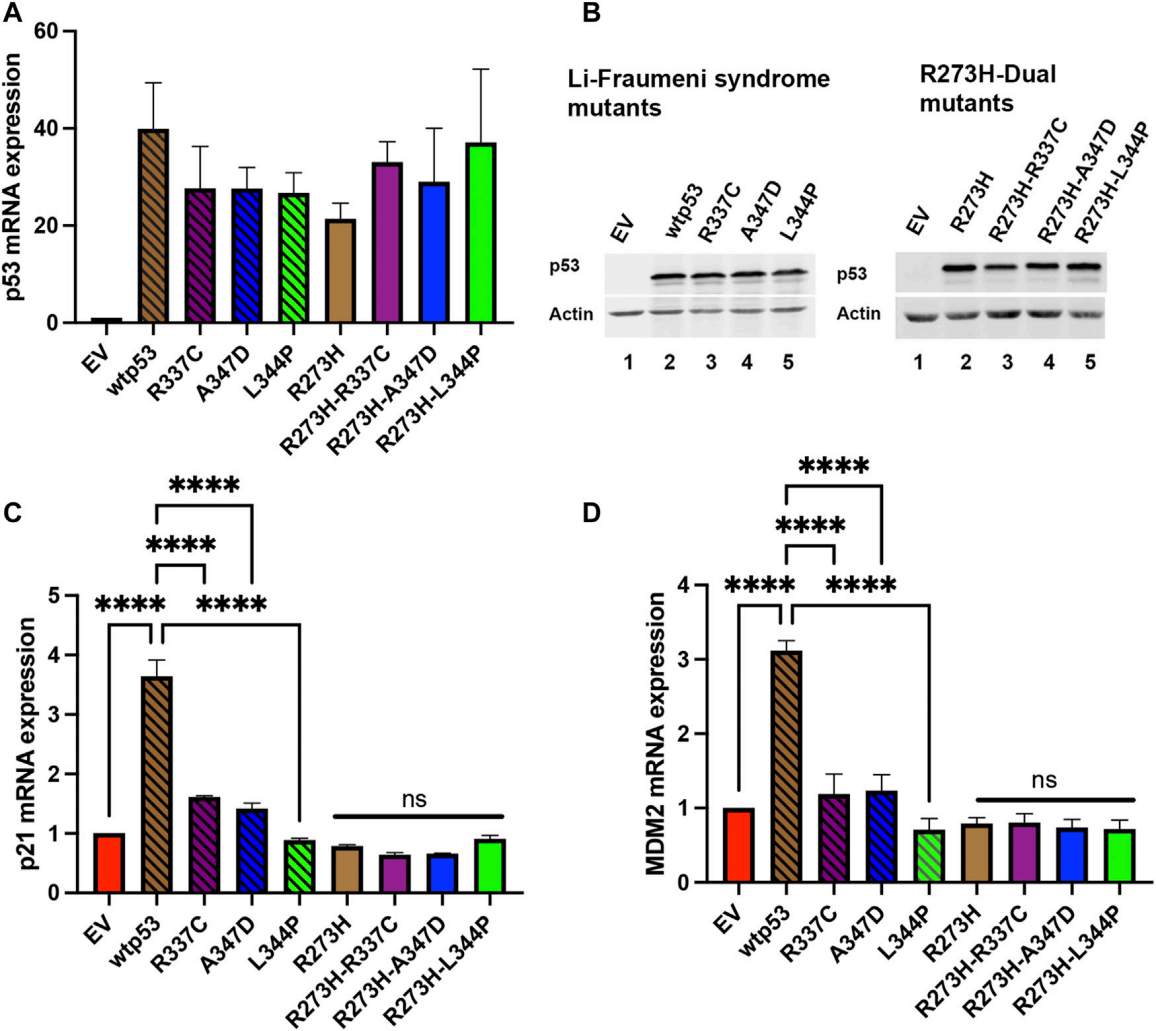
Both *p21* and *MDM2* are activated by wtp53 but not mtp53. HCT116 p53−/− cells transfected with plasmids expressing wtp53, or single mutants R337C, A347D or L344P; or mtp53 R273H, or dual mutants R273H-R337C, R273H-A347D, or R273H-L344P were harvested 24 h post-transfection. The pellets were divided into two and used for either protein or RNA extraction. RNA was extracted from pellet and 5 μg of RNA used for cDNA synthesis. The TaqMan real-time PCR was used to measure the mRNA expression levels of *p53* **(A)**, *p21* **(C)** and *MDM2* **(D)** target genes using *GAPDH* as endogenous control. The data represent an average of three independent biological replicates. A one-way ANOVA was used to determine the statistical significance of the data. The following format was used to assign significance based on *p*-value: **** represents a *p*-value ≤ 0.0001 and ns represent non-significant. **(B)** Pellets were lysed in RIPA buffer and 25 μg of lysate loaded on a 10% SDS-PAGE and probed with anti-p53 DO1 antibody. Actin was used as a normalizer. Data represent three independent biological replicates.

The p53 protein activates the transcription of the cyclin-dependent kinase *p21* and the p53 inhibitor *MDM2* (Levine, 1997; Wu and Levine, 1997). We wanted to confirm that destabilization of wtp53 oligomerization blocked transactivation. We expressed the R337C, A347D, or L344P single or dual mtp53 R273H in HCT116 p53−/− cells and observed significant *TP53* message and protein expression in all the transfection experiments (Figures 2A,B, compare empty vector (EV) to expression constructs as indicated). As expected, upregulation of the endogenous targets *p21* and *MDM2* occurred in the presence of wtp53 expression (Figures 2C,D), and the LFS mutations blocked the ability of p53 to transactivate *p21* and *MDM2* (Figures 2C,D). Equally as predictable, the mtp53 R273H mtp53 expression was unable to activate expression of p*21* or *MDM2*, and dual mutants were no different (Figures 2C,D). The mtp53 R273H has been shown to activate transcriptional targets *CDC7* and *RRM2* (Kollareddy et al., 2015; Datta et al., 2017). As such, we examined the amount of mRNA and protein expression levels of these targets. We examined whether mtp53 R273H, or the mtp53 R273H dual mutants, significantly upregulated the expression of either *CDC7* or *RRM2* (Supplementary Figure S2). However, we did not detect upregulation for these genes. This could be due to low transfection efficiency (30% efficiency), or other variables that may have influenced mtp53 mediated transcription effects.

### Destabilizing Oligomerization of mtp53 Does Not Block mtp53 Interaction With Chromatin

**FIGURE 3:**
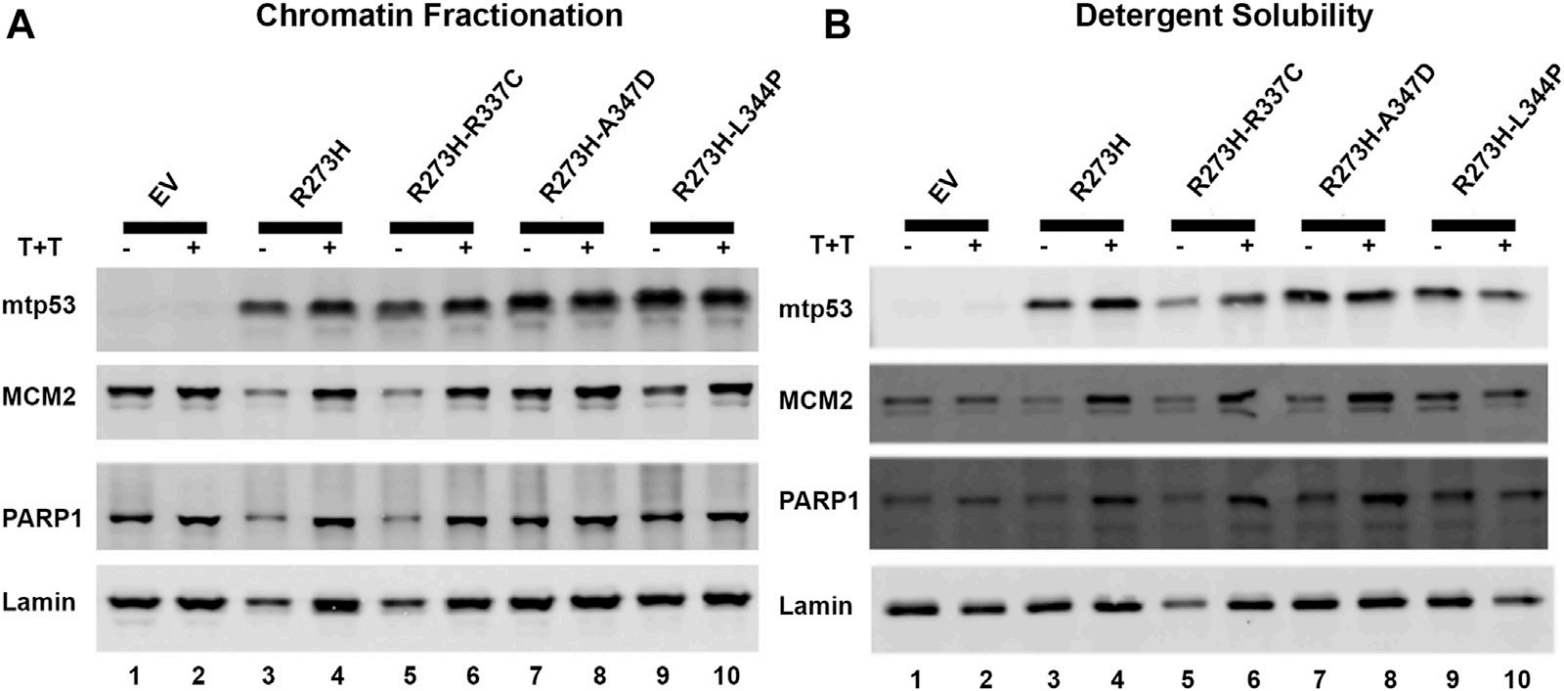
Destabilizing oligomerization of mtp53 R273H does not block the interaction with chromatin. The chromatin fractionation and the detergent solubility assays were used to fractionate lysates prepared from HCT116 p53−/− cells transfected with mtp53 R273H, or dual mutants R273H-R337C, R273H-A347D, or R273H-L344P into cytosolic/soluble fraction and chromatin/insoluble fraction. Analysis of lysates from cells without drug treatment (lanes 1,3,5,7,9) or with a combination of temozolomide (T) at 1 mmol/L plus talazoparib (T) at 10 μmol/L for 4 h (lanes 2,4,6,8,10) was carried out. Binding of mtp53, MCM2, and PARP1 to chromatin isolated from partially purified lysed nuclei (chromatin fractionation assay) or high detergent total cell lysates (detergent solubility assay) was assessed by western blot analysis. 25 μg of chromatin protein was loaded on 10% SDS-PAGE gel. **(A)** Western blot depicting the chromatin association of p53, MCM2, and PARP1 after chromatin fractionation. **(B)** Western blot analysis depicting the chromatin association of p53, MCM2, and PARP1 after detergent solubility. The protein expression level of each target protein was normalized to lamin. The data represent an average of three independent biological replicates. ImageJ quantification of protein expression level is represented in Supplementary Figures S3A,B.

Endogenous mtp53 R273H, and other mtp53 proteins, tightly tether to chromatin (Polotskaia et al., 2015; Qiu et al., 2017). We tested whether the tetramerization state of mtp53 was crucial for the mtp53 chromatin association. We transiently transfected in mtp53 R273H without, and with, the OD mutations. To further examine how dual mutants influenced chromatin interaction, we treated cells with a combination of the alkylating agent temozolomide (Temo) and the PARP1 trapping drug talazoparib (Tal) to induce replication stress and trap PARP1 on the chromatin (Qiu et al., 2017; Xiao et al., 2020). We then isolated chromatin by either chromatin fractionation, or the more stringent detergent insoluble fractionation assay, as methods to assess protein-DNA tethering. We observed mtp53 R273H and mtp53 R273H-dual oligomerization mutants tightly tethered to chromatin (Figures 3A,B, with imageJ quantification shown in Supplementary Figures S3A,B). The replication stress did not significantly alter the ability of mtp53 to interact with chromatin regardless of the OD domain status (Figures 3A,B; Supplementary Figures S3A,B). We therefore conclude that oligomerization of mtp53 R273H is not required for mtp53 chromatin association. Moreover, both PARP and MCM2 interacted well with the chromatin in both unstressed and stressed conditions. The p53 protein that cannot form tetramers does not activate gene transcription. However, the possibility exists that monomers and dimers are able to maintain non-specific chromatin interactions. We compared wtp53, with and without OD mutations, for their general chromatin interactions by transfecting HCT116 p53 −/− cells with plasmids expressing wtp53 or the single OD mutants. Western blot analysis of the chromatin fractionated samples demonstrated that the different oligomerization forms tethered well to the chromatin. As such, mutation in the OD of p53 disrupts transcription factor function but does not disrupt the ability of the protein to generally interact with chromatin (Supplementary Figure S3C).

### Destabilizing Oligomerization of R273H mtp53 Does Not Inhibit Interaction Between mtp53 and MCM2

**FIGURE 4:**
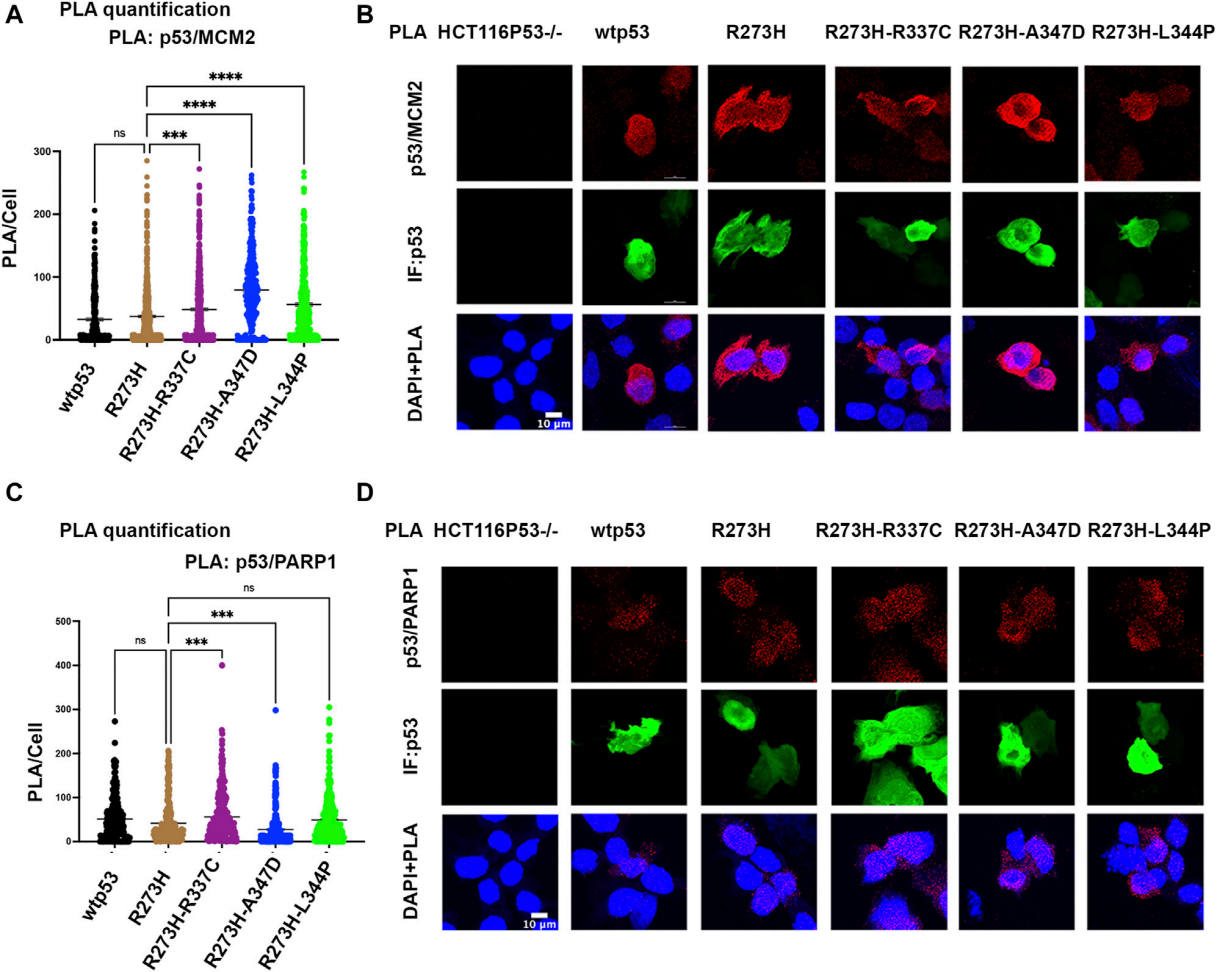
Destabilizing oligomerization of R273H mtp53 does not block the interaction between mtp53 and MCM2. HCT116 p53−/− cells were transfected with wtp53, mtp53 R273H, or dual mutants R273H-R337C, R273H-A347D, R273H-L344P expressing plasmids. **(A)** Analysis of p53/MCM2 complexes by *in situ* proximity ligation assay (PLA). Fluorescent foci per cell were counted using Cellprofiler software and depicted as a scatter plot using GraphPad Prism 9. The data represent a scatter plot with n = 3. An ordinary one-way ANOVA was used to determine the statistical significance of the data. The following format was used to assign significance based on *p*-value: **** represents a *p*-value ≤ 0.0001 and ns represent non-significant. **(B)** Representative confocal microscope images of p53/MCM2 complexes (red) by *in situ* proximity ligation assay (PLA), p53 expression (green) by immunofluorescence microscopy. DNA was counterstained with DAPI (blue). The z-stack maximum intensity projection images are shown. Three independent experiments were performed. (Scale bar = 10 µm). **(C)** Analysis of p53/PARP1 complexes by *in situ* proximity ligation assay (PLA). Fluorescent foci per cell were counted using Cellprofiler software and depicted as a scatter plot using GraphPad Prism 9. The data represent a scatter plot with n = 3. An ordinary one-way ANOVA was used to determine the statistical significance of the data. The following format was used to assign significance based on *p*-value: **** represents a *p*-value ≤ 0.0001 and ns represent non-significant. **(D)** Representative confocal microscope images of p53/PARP1 foci/complexes (red) by *in situ* proximity ligation assay (PLA), p53 expression (green) by immunofluorescence microscopy. DNA was counterstained with DAPI (blue). The z-stack maximum intensity projection images are shown. Three independent experiments were performed. (Scale bar = 10 µm).

We tested if oligomerization influenced the interaction of mtp53 R273H with either MCM2 or PARP1 by using the proximity ligation assay (PLA), which was previously used to demonstrate their endogenous interactions (Xiao et al., 2020). We used transfected HCT116 p53−/− cells in order to compare the interactions with similar levels of exogenously expressed protein for wtp53 and R273H mtp53 single and dual mutants. The expression of GFP was used as a marker for cells expressing p53 and importantly PLA foci were only detected in p53 expressing cells. Previously we had detected a low number of PLA foci for wtp53 in an endogenously expressing cell line. This was due to the fact that endogenous wtp53 protein is maintained at low levels in MCF7 cancer cells due to degradation by the E3 ubiqutin ligase MDM2 (Xiao et al., 2020). Interestingly, when the expression level was the same we detected similar interaction levels between MCM2 with both wtp53 and mtp53 R273H (Figures 4A,B). In contrast, the dual mtp53 R273H-OD mutants demonstrated slightly more MCM2-mtp53 associated foci (Figures 4A,B). The interaction between mtp53 and PARP1 was also maintained after introducing mutations to destabilize tetramer formation, with no observable increase for the dual mutants (Figures 4C,D). This suggests that R273H hotspot mtp53 may interact with replicating DNA in forms that are not tetrameric. We also assessed the single-OD mutants and observed interactions between the single-OD p53 mutants and MCM2 as well as PARP1 (Supplementary Figure S4). This further supports the possibility that non-tetramerized p53 may function in alternative, non-transcription related, pathways that mediate GOF activity.

### Deletion of the Non-specific DNA Binding Domain of mtp53 Decreases the Interaction Between mtp53 R273H and MCM2

**FIGURE 5:**
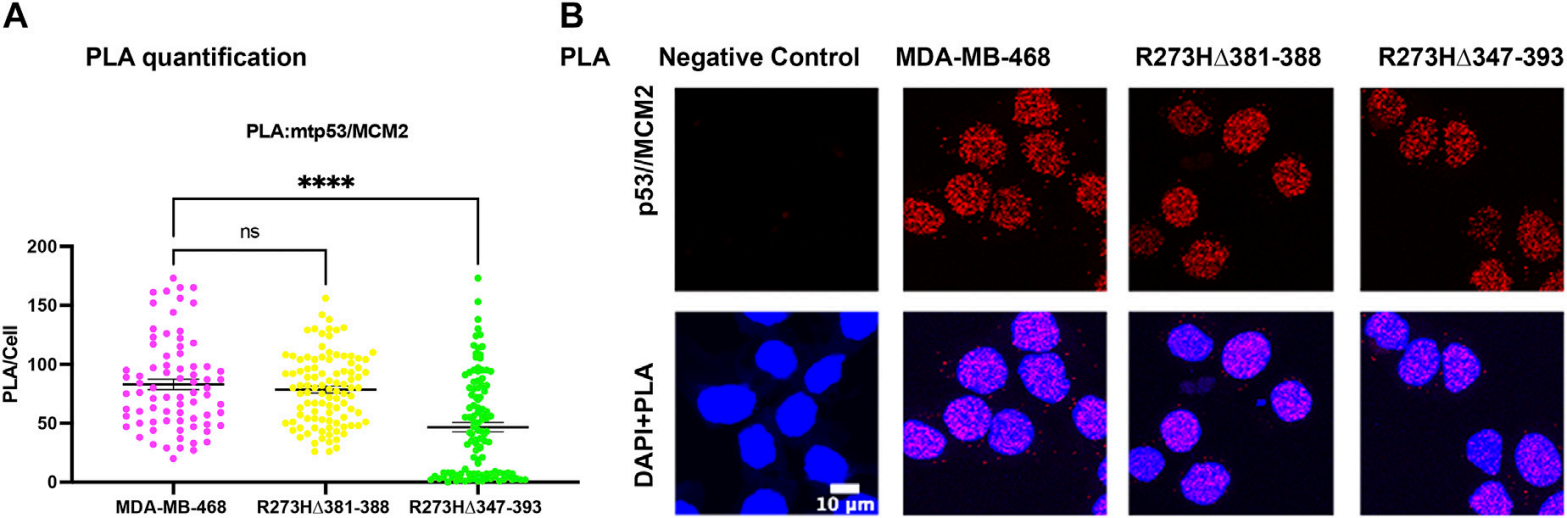
Endogenous deletions in the OD of R273H mtp53 decreases the interaction between mtp53 and MCM2. **(A)** Analysis of p53/MCM2 complexes by *in situ* proximity ligation assay (PLA) in MDA-MB-468, R273HΔ381-388 and R273HΔ347-393 cells. Fluorescent foci per cell were counted by Cellprofiler software and depicted by a scatter plot using GraphPad Prism 9. The data represent a scatter plot with n = 3. An ordinary one-way ANOVA was used to determine the statistical significance of the data. The following format was used to assign significance based on *p*-value: **** represents a *p*-value ≤ 0.0001 and ns represent non-significant. **(B)** Representative confocal microscope images of p53/MCM2 foci/complexes (red) by *in situ* proximity ligation assay (PLA) in MDA-MB-468, R273HΔ381-388 and R273HΔ347-393 cells. DNA was counterstained with DAPI (blue). The z-stack maximum intensity projection images are shown. Three independent experiments were performed. (Scale bar = 10 µm).

Generation of R273H mtp53 deletions of a small portion of the C-terminus (R273HΔ381-388) and a larger deletion removing some of the OD and all of the C-terminus (R273HΔ347-393) in MDA-MB-468 cells causes replication stress (Ellison et al., 2021). The larger deletion R273HΔ347-393 dual mutant causes drastic inhibition of cell proliferation. As such, we wondered if the entire deletion of the C-terminal non-specific DNA binding domain R273HΔ347-393 inhibited the interaction of mtp53 R273H with the replication machinery. We tested the interaction between mtp53 and MCM2 using the PLA method and observed that R273HΔ347-393, but not the smaller deletion R273HΔ347-393, exhibited decreased interaction with the MCM2 replication helicase (Figures 5A,B).

## Discussion

Wild-type p53 requires tetramization to be a functional transcription factor but oncogenic mtp53 does not act as a direct transcription factor. As such, it remains to be determined what roles are played by the different oligomerization forms of oncogenic mutant p53. The newly generated dual mutants have both a DNA binding domain mutation and an oligomerization domain mutation. Dual mutants are not naturally occurring in cancers. Mutations in the OD that disrupt p53 transcription factor function may allow for p53 functions (that to date have not been discovered and/or described) to be co-opted in the mutant p53 isoforms. It is possible that monomers of p53 may have a transcription-independent chromatin associated function, and our work may provide clues for how equilibrium between different p53 oligomerization forms influence different cellular functions. The observation of altered tumor-suppressor function resulting from LFS mutations within the OD is similar to that observed in hotspot DNA binding domain mutants (Levine, 2021). Both types of mutations lead to a loss of function for wtp53 tumor suppressor functions. In the resulting cancers high levels of stable mtp53 occur (Kim and Lozano, 2018). We have explored how dual region mutations, in both the DNA binding domain and OD, influence the mtp53 replication-associated chromatin functions. We were able to change the oligomerization state of mtp53 R273H such that the R273H-dual mutants possessed similar oligomerization to their corresponding single LFS mutants (Figure 1).

The LFS mutations R337C, A347D, and L344P within the OD as expected blocked the transactivation of wtp53 (Figure 2). The DNA binding domain mutation R273H compromised the ability of the protein to activate transcription from endogenous p53 responsive elements. In the case of the mtp53 R273H-dual mutants, the presence of an extra mutation that prevented tetramer formation, not surprisingly, had no influence on the transactivation ability (Figure 2). We did not observe mtp53 R273H mediated transactivation of either *CDC7* or *RRM2* (Supplementary Figure S2). This supports the recent finding that outcomes on activation of new genes may result from aneuploidy and are not direct results of mtp53 R273H transactivation (Redman-Rivera et al., 2021).

Chromatin tethering of mtp53 has implications in DNA replication and repair mechanisms. Prior to this study, various reports confirmed that most of the chromatin association of wtp53 occurs as a tetramer (McLure and Lee, 1998; McLure and Lee, 1999; Weinberg et al., 2004). We observed that hotspot GOF mtp53 R273H can form tetramers in cancer cell lines (Supplementary Figure S1) but that tetramerization is not required for chromatin interaction, or interaction with MCM2 (Figures 3, 4). The DNA replication machinery is under the regulation of protein complexes that control origin licensing, firing, unwinding, and relaxation (Klusmann et al., 2016). GOF mtp53 has been implicated in a variety of DNA replication processes, including an increase in replication origin firing and enabling the interaction between TopBP1 with Treslin to induce Cdk2 (Kollareddy et al., 2015; Singh et al., 2017). We previously reported a close association between mtp53, replicating DNA, and PARP1 (Xiao et al., 2020). Herein, we explored whether the oligomerization of mtp53 R273H was required for it to interact with PARP1, and the replication helicase MCM2. Our results suggest that the interaction between mtp53 R273H and PARP is not significantly altered by destabilizing mtp53 tetramer formation. On the other hand, there was a slight increase in the interaction between mtp53 R273H-dual OD mutants and MCM2 (Figure 4). This suggests that non-tetrameric forms of p53 may interact more often with the DNA replication machinery. Interestingly, the absence of the entire C-terminal domain (which is involved in non-specific DNA binding and nuclear localization) in the R273HΔ347-393 mutant (Ellison et al., 2021), had a reduced interaction with MCM2 (Figure 5). Taken together, the data presented here showed that oncogenic mtp53 R273H can form tetramers, but that the dynamics of tetramer formation and the C-terminal non-specific DNA binding domain may differentially regulate the GOF replication-associated activities. We are in the process of carrying out further experiments to explore how destabilizing tetramer formation and non-specific DNA binding influence the association between mtp53 R273H and replicating DNA.

## Data Availability

The raw data supporting the conclusion of this article will be made available by the authors, without undue reservation.
