# Supplementary figures and images for: Oligomerization of Mutant p53 R273H is not Required for Gain-of-Function Chromatin Associated Activities
Source: Front Cell Dev Biol. 2021 Nov 22;9:772315. doi: 10.3389/fcell.2021.772315 (PMC8645790; doi:10.3389/fcell.2021.772315)

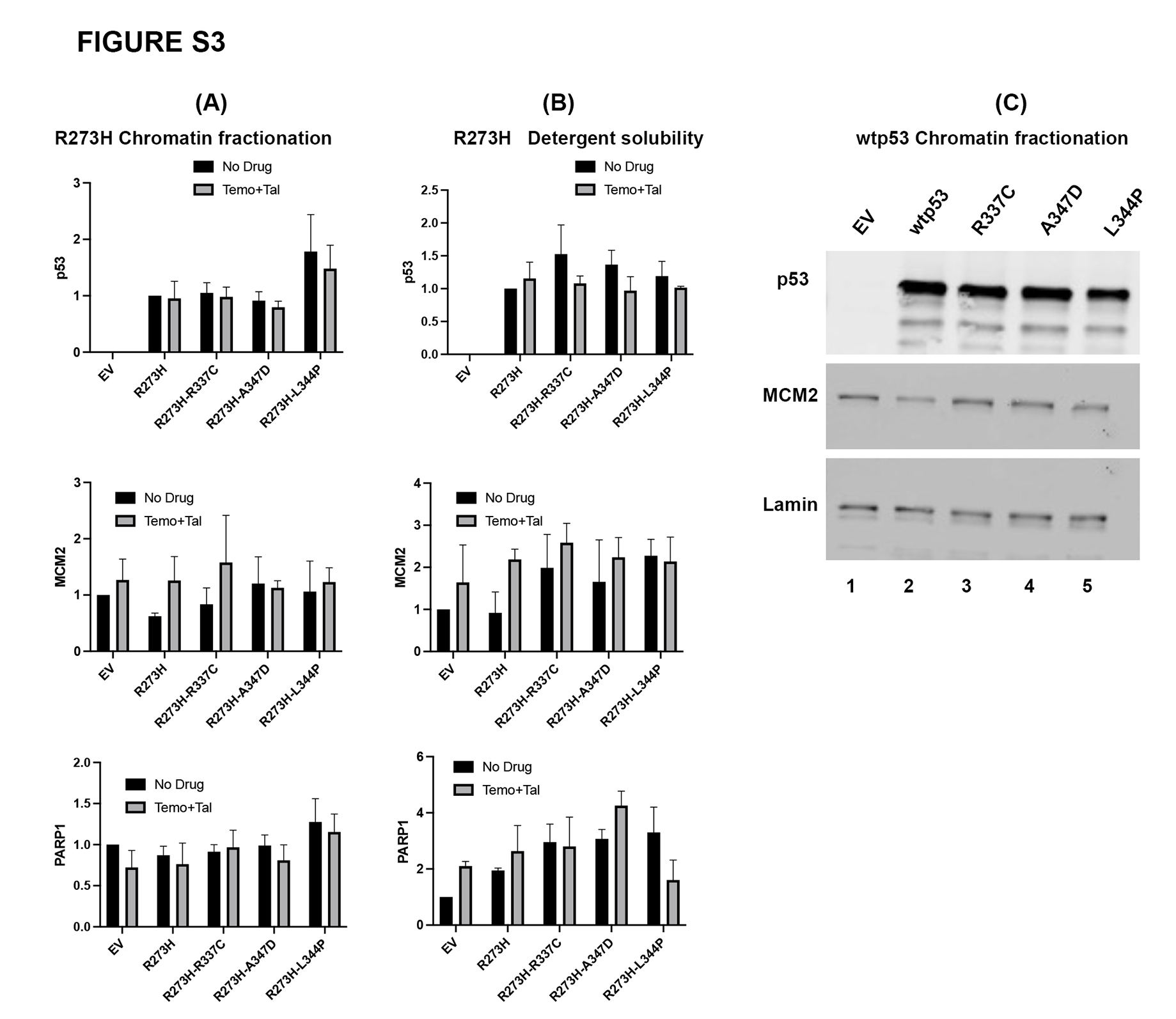

Supplement: Supplementary file 1 [file Image3.TIF]

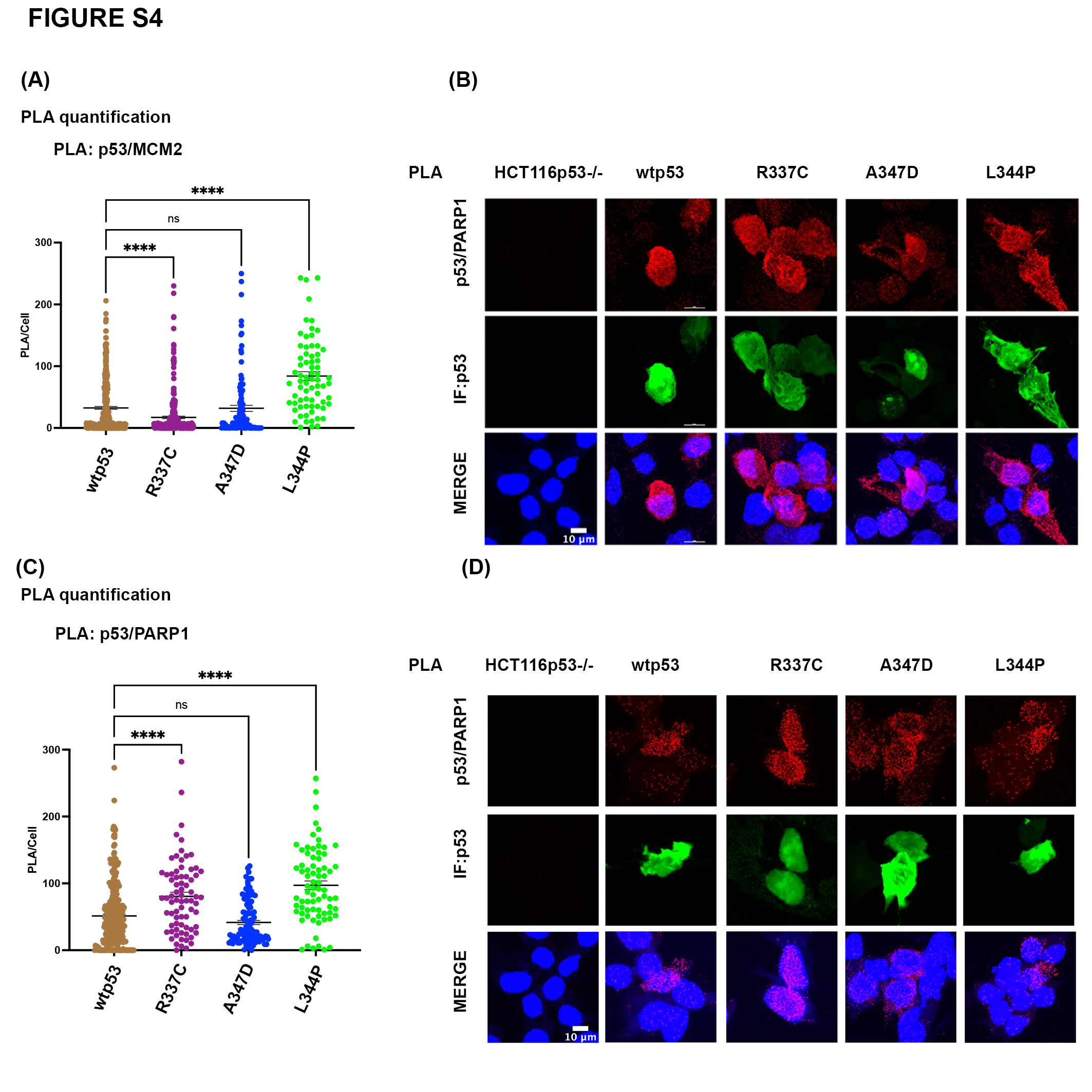

Supplement: Supplementary file 2 [file Image4.TIF]

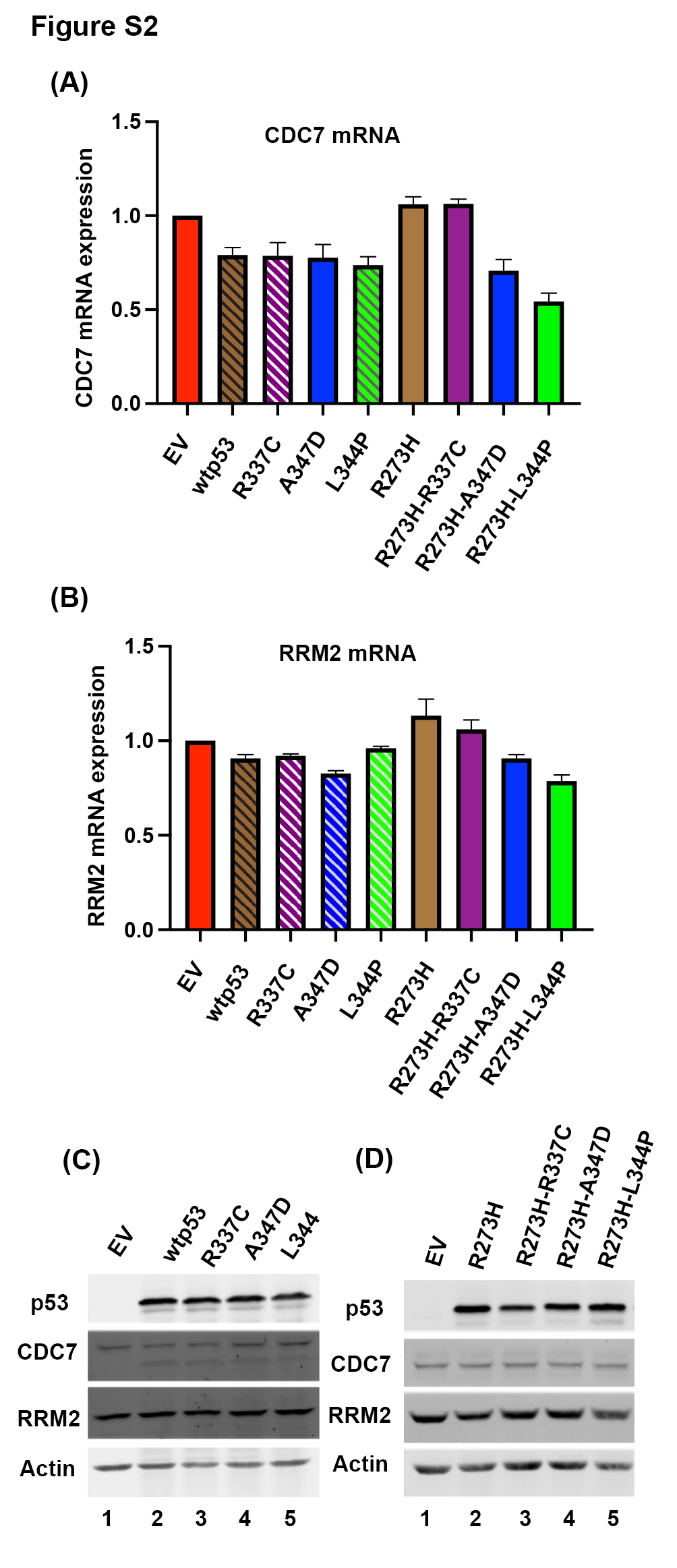

Supplement: Supplementary file 3 [file Image2.TIF]

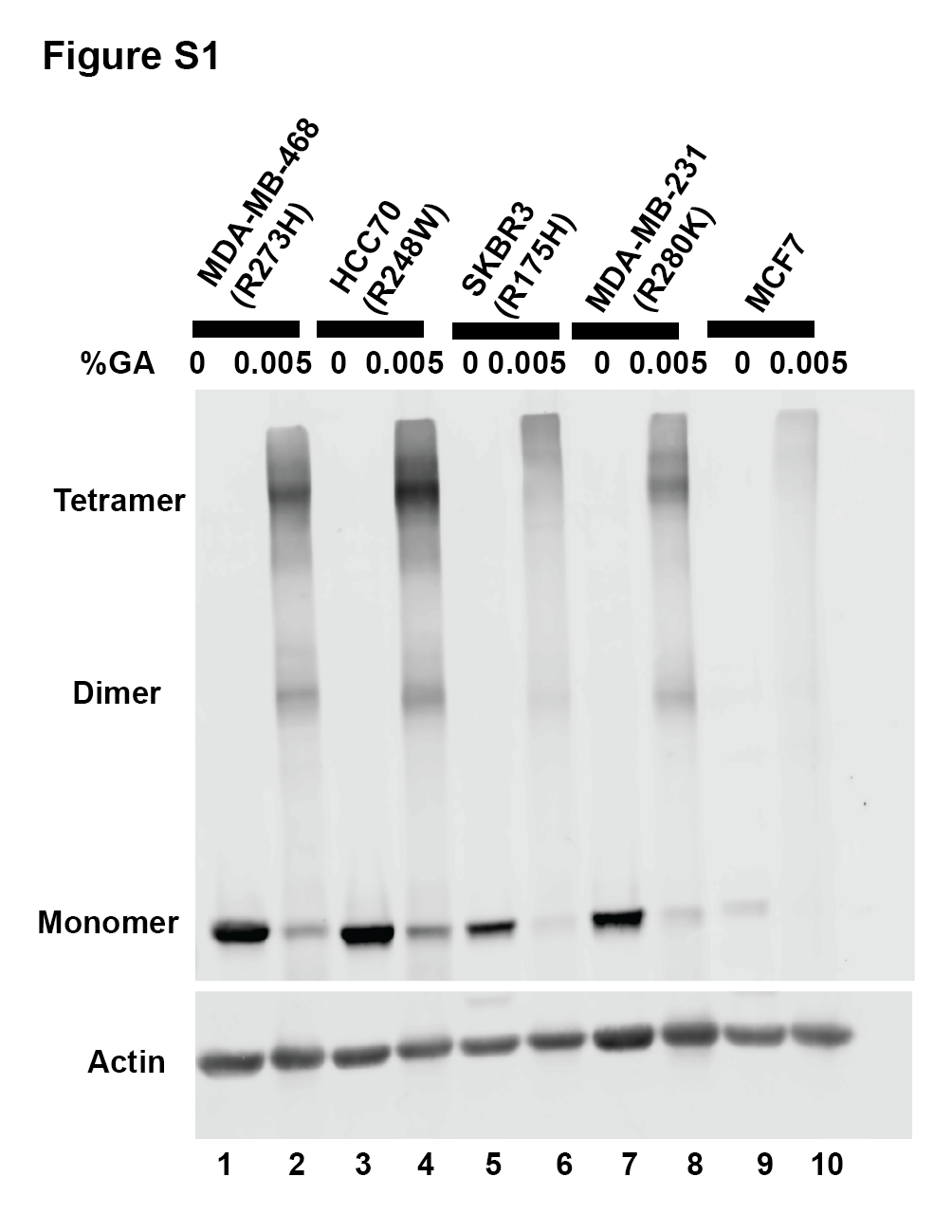

Supplement: Supplementary file 4 [file Image1.TIF]
