# Supplementary material for: Oligomerization of Mutant p53 R273H is not Required for Gain-of-Function Chromatin Associated Activities
Source: Front Cell Dev Biol. 2021 Nov 22;9:772315. doi: 10.3389/fcell.2021.772315 (PMC8645790; doi:10.3389/fcell.2021.772315)
Supplement: Supplementary file 5 [file DataSheet1.DOCX]

**Figure S1: Gain-of-Function hotspot mtp53 in cancer cells predominantly forms tetramers**.

Cells were harvested at 70-90% confluency by scraping and washing with cold PBS followed by lysing. 50 μg of the resulting cell lysates were treated with either 0% (lanes 1,3,5,7,9) or 0.005% (lanes 2,4,6,8,10) glutaraldehyde for 20 min at room temperature. Samples were run on an 8% SDS-PAGE and oligomerization was determined by western blotting using anti-p53 DO1 antibody. Actin was used as a normalizer and showed a very minor shift in mobility with 0.005% glutaraldehyde. Data presented was reproduced in 3 biological replicates

**Figure S2: Transactivation of *CDC7* and *RRM2* is not upregulated by mtp53.** HCT116 p53-/- cells transfected with plasmids expressing wtp53, or single mutants R337C, A347D or L344P; or mtp53 R273H, or dual mutants R273H-R337C, R273H-A347D, or R273H-L344P. Cells were harvested 24 hr post-transfection. The pellets were divided into two and used for either protein or RNA extraction. RNA was extracted from the cell pellet and 5 μg of RNA used for cDNA synthesis. The mRNA message of *CDC7* **(A)**, and *RRM2* **(B)** was measured by TaqMan qRT-PCR. The data represent an average of 3 independent biological replicates. A one-way ANOVA was used to determine the statistical significance. No statistically significant upregulation was observed. Wtp53, or single mutants R337C, A347D or L344P **(C)** and mtp53 R273H, or dual mutants R273H-R337C, R273H-A347D, or R273H-L344P **(D)** were cells were lysed in RIPA buffer and 25 μg of the lysate was loaded on a 10% SDS-PAGE and probed with anti-p53 DO1, anti-CDC7 and anti-RRM2 antibodies. Actin was used as a normalizer. Data represent 3 independent biological replicates. The p53 western blot images are also shown in Figure 2.

**Figure S3: Destabilizing oligomerization of wtp53 does not block the interaction with chromatin.** The protein expression levels of mtp53, MCM2 and PARP1, normalized to lamin were quantified using ImageJ. A graphical representation of the protein expression for **(A)** chromatin fractionation and **(B)** detergent solubility assays are shown. The date represents three independent biological replicates. (**C)** The chromatin fractionation assay was used to examine HCT116 p53-/- cells transfected with wtp53, or single mutants R337C, A347D, or L344P into the cytosolic/soluble fraction and the chromatin/insoluble fraction. Binding of mtp53 and MCM2, to chromatin isolated from partially purified lysed nuclei was assessed by western blot analysis. 25 μg of chromatin protein was loaded on a 10% SDS-PAGE gel. The western blot shows the chromatin association of p53 and MCM2. Data presented was reproduced in 2 biological replicates

**Figure S4: Destabilizing oligomerization of wtp53 (mutations made by site directed mutagenesis) does not block the interaction between mtp53 and MCM2.** HCT116 p53-/- cells were transfected with wtp53, or single mutants R337C, A347D, or L344P expressing plasmids. **(A)** Analysis of p53/MCM2 complexes by in situ proximity ligation assay (PLA). Fluorescent foci per cell were counted using Cellprofiler software and depicted as a scatter plot using GraphPad Prism 9. The data represent a scatter plot with n=3. An ordinary one-way ANOVA was used to determine the statistical significance of the data. The following format was used to assign significance based on P-value: **** represents a p-value ≤ 0.0001 and ns represent non-significant. **(B)** Confocal microscope images of p53/MCM2 complexes (red) by in situ proximity ligation assay (PLA), p53 expression (green) by immunofluorescence microscopy. DNA was counterstained with DAPI (blue). The z-stack maximum intensity projection images are shown. Three independent experiments were performed. (Scale bar = 10µm). **(C)** Analysis of p53/PARP1 complexes by in situ proximity ligation assay (PLA). Fluorescent foci per cell were counted using Cellprofiler software and depicted as a scatter plot using GraphPad Prism 9. The data represent a scatter plot with n=3. An ordinary one-way ANOVA was used to determine the statistical significance of the data. The following format was used to assign significance based on P-value: **** represents a p-value ≤ 0.0001 and ns represent non-significant. **(D)** Confocal microscope images of p53/PARP1 complexes (red) by in situ proximity ligation assay (PLA), p53 expression (green) by immunofluorescence microscopy. DNA was counterstained with DAPI (blue). The z-stack maximum intensity projection images are shown. Three independent experiments were performed. (Scale bar = 10µm).
